# Potentiation of BK_Ca_ channels by cystic fibrosis transmembrane conductance regulator correctors VX-445 and VX-121

**DOI:** 10.1172/JCI176328

**Published:** 2024-07-02

**Authors:** Aaron Kolski-Andreaco, Stefanie Taiclet, Michael M. Myerburg, John Sembrat, Robert J. Bridges, Adam C. Straub, Zachary P. Wills, Michael B. Butterworth, Daniel C. Devor

**Affiliations:** 1 Department of Cell Biology,; 2 Department of Pharmacology and Chemical Biology, and; 3 Division of Pulmonary, Allergy, Critical Care and Sleep Medicine, University of Pittsburgh, Pittsburgh, Pennsylvania, USA.; 4 Department of Physiology and Biophysics, Chicago Medical School, North Chicago, Illinois, USA.; 5 Department of Neurobiology, University of Pittsburgh, Pittsburgh, Pennsylvania, USA.

**Keywords:** Therapeutics, Chloride channels, Drug therapy, Potassium channels

## Abstract

Cystic fibrosis results from mutations in the cystic fibrosis transmembrane conductance regulator (CFTR) anion channel, ultimately leading to diminished transepithelial anion secretion and mucociliary clearance. CFTR correctors are therapeutics that restore the folding/trafficking of mutated CFTR to the plasma membrane. The large-conductance calcium-activated potassium channel (BK_Ca_, K_Ca_1.1) is also critical for maintaining lung airway surface liquid (ASL) volume. Here, we show that the class 2 (C2) CFTR corrector VX-445 (elexacaftor) induces K^+^ secretion across WT and F508del CFTR primary human bronchial epithelial cells (HBEs), which was entirely inhibited by the BK_Ca_ antagonist paxilline. Similar results were observed with VX-121, a corrector under clinical evaluation. Whole-cell patch-clamp recordings verified that CFTR correctors potentiated BK_Ca_ activity from both primary HBEs and HEK cells stably expressing the α subunit (HEK-BK cells). Furthermore, excised patch-clamp recordings from HEK-BK cells verified direct action on the channel and demonstrated a significant increase in open probability. In mouse mesenteric artery, VX-445 induced a paxilline-sensitive vasorelaxation of preconstricted arteries. VX-445 also reduced firing frequency in primary rat hippocampal and cortical neurons. We raise the possibilities that C2 CFTR correctors gain additional clinical benefit by activation of BK_Ca_ in the lung yet may lead to adverse events through BK_Ca_ activation elsewhere.

## Abstract 2

C2 CFTR correctors are potent activators of BKCa that promote potassium secretion and vasorelaxation and affect neuronal firing properties.

## Graphical abstract

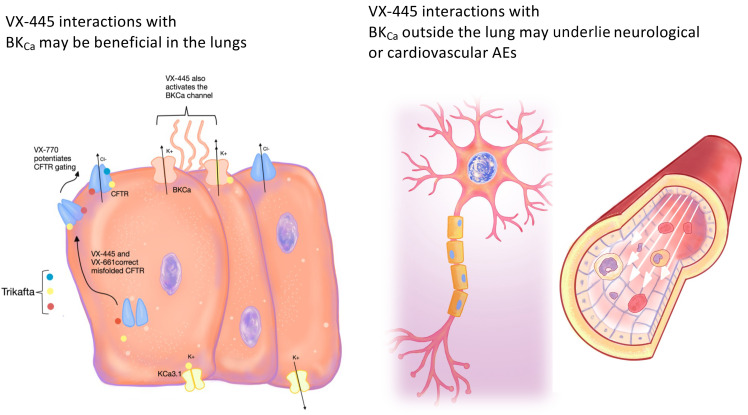

## Introduction

Cystic fibrosis (CF) affects approximately 40,000 individuals in the United States and approximately 100,000 people worldwide (1, 2). The pathogenesis of CF is the result of mutations to the cystic fibrosis transmembrane conductance regulator (CFTR) protein, an apical membrane anion channel in epithelia. In the human airway, dysfunctional anion secretion leads to a reduction in airway surface liquid (ASL) height, resulting in reduced mucociliary clearance and an increase in the risk of infection (3–6). CF can be caused by one of roughly 1,700 known mutations to the CFTR gene, which are separated into 6 unique categories based on how they result in a loss of CFTR expression/function (7). The 2 most common categories of mutations include those that affect the trafficking and gating of CFTR. F508del is the most common folding mutation, occurring in approximately 85% of patients with CF, and results in an anion channel that fails to correctly traffic to the apical membrane. Therapeutics that target folding/trafficking mutations are known as “correctors.” Corrector compounds can restore plasma membrane expression of F508del; however, this mutated CFTR protein is still incapable of gating correctly, so additional therapeutics are required, termed “potentiators,” which restore CFTR function (8, 9).

Over the past 15 years, significant advances have been made in CF therapeutics. VX-770 (ivacaftor) emerged as the first clinically approved potentiator therapy for CF (8) — a drug that increases CFTR open probability (P_o_). More recently, CFTR correctors, including the class 1 (C1) corrector VX-661 (tezacaftor) and the class 2 (C2) corrector VX-445 (elexacaftor), were developed that exhibit unique mechanisms of action and binding sites on CFTR (10–16). In 2019, the combination of elexacaftor, tezacaftor, and ivacaftor (ETI) was approved by the FDA as the highly effective modulator therapy (HEMT) Trikafta (17, 18). This HEMT made it possible to effectively treat people with CF who have at least 1 F508del allele by improving lung function and hence quality of life (18, 19).

In addition to apical CFTR, transepithelial anion secretion requires the coordinated regulation of multiple conductances, including basolateral K^+^ channels, which are responsible for potassium recycling and maintenance of the electrochemical driving force for apical Cl^–^ efflux. Our group demonstrated that pharmacological activation of a basolateral Ca^2+^-activated K^+^ conductance (KCa3.1) stimulates Cl^–^ secretion across a wide array of epithelia, including human bronchial epithelial cells (HBEs) (20–25). In the apical membrane, the BK_Ca_ (KCa1.1) channel has been shown to promote Cl^–^ secretion, and hence regulate ASL volume in HBEs. Indeed, pharmacological inhibition of BK_Ca_ channels significantly reduces ASL volume (26). Importantly, the CF-associated inflammatory mediators IFN-γ and TGF-β decrease BK_Ca_ channel expression, which correlates with a reduction in ASL volume (27, 28). Based on these data, it has been proposed that pharmacological activation of BK_Ca_ may be therapeutically beneficial in CF.

Given the role of BK_Ca_ in modulating transepithelial Cl^–^ secretion, we determined the effect of CFTR correctors on K^+^ secretion across HBEs. We demonstrate, for the first time to our knowledge, that the C2 correctors VX-659 (bamocaftor), VX-445, and VX-121 (vanzacaftor) potentiate K^+^ secretion across WT and F508del CFTR–expressing HBEs via Ussing chamber short-circuit current experiments. This C2-mediated K^+^ secretion was entirely abrogated by the BK_Ca_ channel antagonists paxilline and iberiotoxin (IBTX). Patch-clamp studies verified that C2 correctors potentiate BK_Ca_ both heterologously expressed in HEK cells and endogenously expressed in primary HBEs via an increase in channel P_o_. Furthermore, we show that C2 correctors induce vasorelaxation of microvascular arteries and significantly alter neuronal excitability — two effects consistent with activation of BK_Ca_ channels. Thus, while C2 corrector–dependent potentiation of BK_Ca_ may be of benefit in airway epithelia, the cross-reactivity with BK_Ca_ in other tissues may contribute to the adverse events reported by patients with CF upon initiation of ETI (29–34).

## Results

In the human airway, activation of basolateral KCa3.1 promotes transepithelial Cl^–^ secretion across WT and corrected F508del HBEs (21, 22, 24, 25, 35). Further, apical BK_Ca_ plays a vital role in maintaining the ASL volume (26–28). Thus, we determined whether CFTR correctors would alter K^+^ channel function when acutely applied to HBEs.

**Figure 1:**
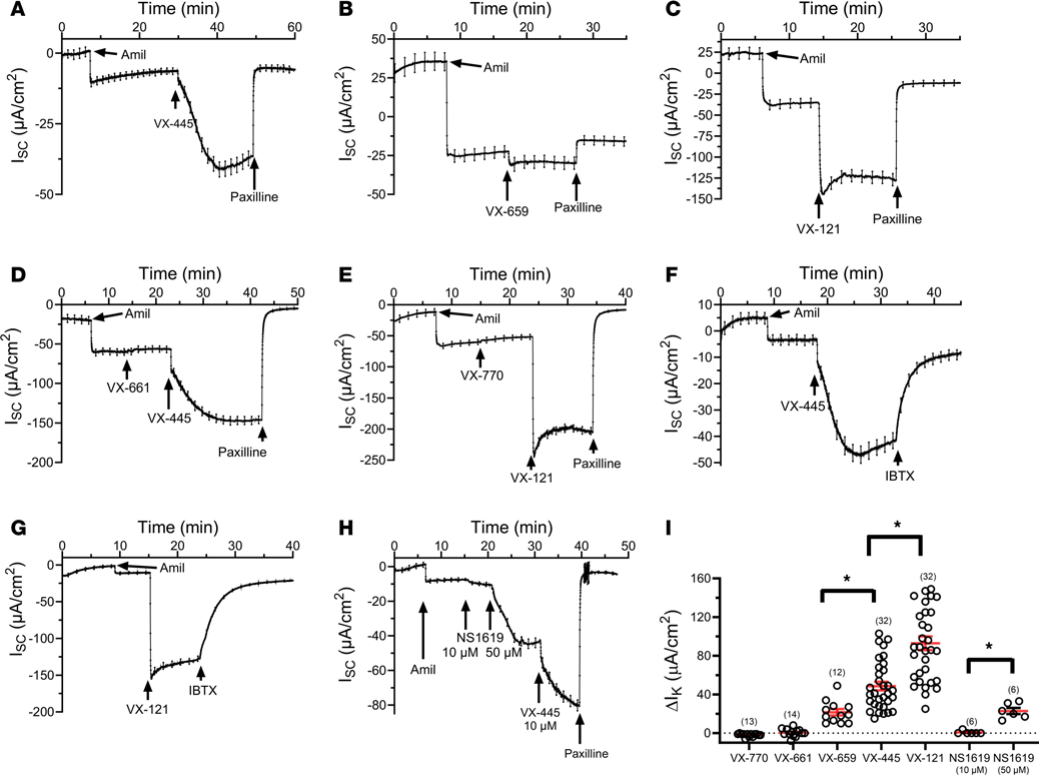
VX-445, VX-659, and VX-121 stimulate BK_Ca_ currents across WT CFTR HBEs. Currents were recorded with a 125:5 mM K^+^ gradient (basolateral to apical). (**A**–**C**) Subsequent to amiloride, short-circuit current (I_sc_) was increased by the C2 CFTR correctors VX-445 (**A**, 10 μM), VX-659 (**B**, 10 μM), and VX-121 (**C**, 10 μM). (**D** and **E**) In contrast, the C1 CFTR corrector VX-661 (**D**, 10 μM) and the CFTR potentiator VX-770 (**E**, 10 μM) failed to increase I_sc_. Subsequent addition of either VX-445 (**D**) or VX-121 (**E**) stimulated I_sc_. In all experiments, the current was completely blocked by the specific BK_Ca_ inhibitor paxilline (10 μM). (**F** and **G**) Additional studies verified that both the VX-445–induced (**F**) and the VX-121–induced (**G**) currents were inhibited by the additional specific BK_Ca_ blocker IBTX (300 nM). (**H**) Subsequent to amiloride, 10 μM NS1619 failed to stimulate I_sc_, while further addition of 50 μM NS1619 induced a marked increase. This response was further increased by VX-445 (10 μM) and inhibited by paxilline. (**I**) Average responses (mean ± SEM, *P < 0.01; 1-way ANOVA) are represented as the change in K^+^ current (ΔI_K_). The magnitude of the K^+^ current was calculated as described in Methods. Nine donors were used in these studies. Experimental replicates are indicated in parentheses above each data set.

Initially, we determined the effect of the current standard of care (SOC) C2 CFTR corrector, VX-445, on BK_Ca_ function in primary WT CFTR HBEs grown at an air-liquid interface on Transwell filters. Studies were carried out in Ussing chambers using a 125:5 mM K^+^ gradient (basal to apical) to measure K^+^ secretion across the epithelia, as previously described (20). Note that our solutions result in a large apical-to-basolateral Na^+^ gradient. As shown in Figure 1A, amiloride was used to inhibit the basal Na^+^ absorption, resulting in an inwardly directed current, consistent with K^+^ secretion across the apical membrane. Subsequent addition of VX-445 (10 μM) stimulated a large, slowly developing increase in inward current that was completely inhibited by the specific BK_Ca_ blocker paxilline (10 μM). This result demonstrates that VX-445 stimulates K^+^ secretion across the apical membrane of HBEs. Consistent with activation of BK_Ca_, VX-445 decreased transepithelial resistance (R_te_) from 521 ± 57 Ω∙cm^2^ in the presence of amiloride to 283 ± 18 Ω∙cm^2^, while addition of paxilline increased R_te_ to 1,155 ± 109 Ω∙cm^2^ (*n* = 30). As shown in Figure 1B, a distinct C2 corrector, VX-659 (10 μM), similarly stimulated a paxilline-sensitive K^+^ current. More recently, Vertex Pharmaceuticals developed a next-generation C2 CFTR corrector, VX-121, which is currently undergoing clinical trials (36). As shown in Figure 1C, subsequent to amiloride, VX-121 (10 μM) also stimulated a large, paxilline-sensitive K^+^ secretory current. As above, VX-121 decreased R_te_ from 453 ± 49 Ω∙cm^2^ in the presence of amiloride to 248 ± 17 Ω∙cm^2^, while addition of paxilline increased R_te_ to 691 ± 68 Ω∙cm^2^ (*n* = 29), consistent with BK_Ca_ activation and inhibition, respectively. We next determined whether other components of ETI would stimulate K^+^ secretion across HBEs. Neither the C1 corrector VX-661 (Figure 1D) nor the CFTR potentiator VX-770 (Figure 1E) induced K^+^ secretion, whereas subsequent addition of VX-445 or VX-121 stimulated K^+^ secretion, respectively. To further confirm that this effect was due to apical BK_Ca_ activation, we determined whether the currents induced by VX-445 and VX-121 could be inhibited by an additional selective BK_Ca_ inhibitor, IBTX. As a 37–amino acid peptide, IBTX cannot cross the apical membrane. As shown, both the VX-445–induced (Figure 1F) and VX-121–induced (Figure 1G) K^+^ currents were inhibited by IBTX (300 nM), verifying activation of BK_Ca_. Similar results were observed in 6 experiments each for VX-445 and VX-121. Given these surprising results, we determined the effect of a known BK_Ca_ opener, NS1619, on K^+^ secretion across HBEs. As shown in Figure 1H, 10 μM NS1619 failed to stimulate K^+^ secretion, though the subsequent addition of 50 μM NS1619 stimulated a substantial K^+^ secretory response. Further addition of VX-445 (10 μM) stimulated an additional K^+^ secretory response that was completely inhibited by paxilline. The average responses are shown in Figure 1I, having a rank order of VX-121 > VX-445 > VX-659 (*P < 0.01).

**Figure 2:**
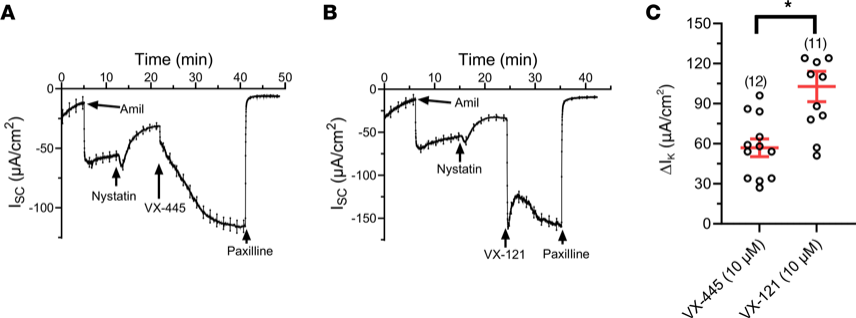
VX-445 and VX-121 stimulate BK_Ca_ currents across WT CFTR HBEs following permeabilization of the basolateral membrane with nystatin. Currents were recorded with a 125:5 mM K^+^ gradient (basolateral to apical). Subsequent to amiloride, nystatin (200 μM) was added to the basolateral membrane. Following establishment of a new stable current, both VX-445 (**A**, 10 μM) and VX-121 (**B**, 10 μM) stimulated an increase in I_K_. (**C**) Average ΔI_K_ (mean ± SEM) for VX-445 and VX-121 (*P < 0.01; unpaired *t* test). ΔI_K_ was calculated as the change in I_K_ between the current in the presence of nystatin and the peak response to VX-445 or VX-121. Three donors were used in these studies. Experimental replicates are indicated in parentheses above each data set.

While the most parsimonious explanation for our results is that C2 correctors potentiate apical BK_Ca_, we cannot rule out a potential role for the basolateral membrane in this response. Therefore, the effects of VX-445 and VX-121 on apical membrane K^+^ currents were assessed after permeabilization of the basolateral membrane with nystatin (200 μM), as previously described (37). Formation of the nystatin pore is visualized in Figure 2, A and B, as a positive shift in baseline current to a new stable plateau. Subsequent addition of either VX-445 (Figure 2A) or VX-121 (Figure 2B) stimulated paxilline-sensitive K^+^ currents that were indistinguishable from those in the absence of permeabilization. Consistent with our intact monolayers, the response to VX-121 was significantly greater than the response to VX-445 (Figure 2C; *P < 0.01). These results verify that C2 CFTR correctors potentiate apical BK_Ca_ in WT CFTR–expressing HBEs.

**Figure 3:**
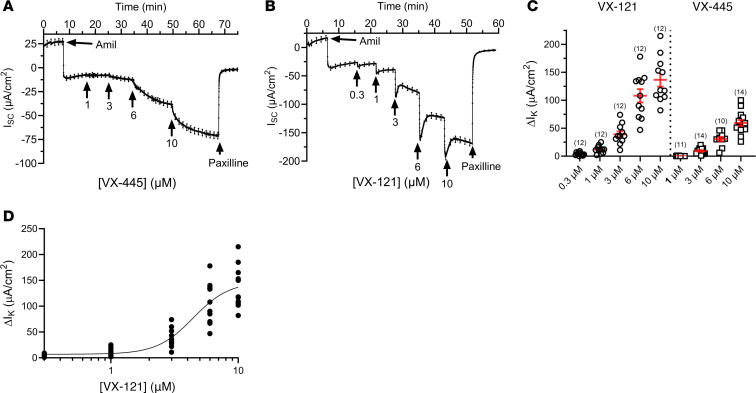
Concentration dependence of VX-445 and VX-121 stimulation of K^+^ current across WT CFTR HBEs. (**A**) Subsequent to amiloride, VX-445 induced a concentration-dependent increase in K^+^ current that was inhibited by paxilline. (**B**) Subsequent to amiloride, VX-121 induced a concentration-dependent increase in K^+^ current that was inhibited by paxilline. (**C**) Average increase in K^+^ current for each concentration of VX-121 and VX-445 (mean ± SEM). Note that for VX-445 there are different numbers of experiments for each concentration, as not all concentrations were used in each experiment. (**D**) Data from **C** for VX-121 were fit to the Hill equation, giving an EC_50_ of 4.4 μM with a Hill coefficient of 3.0 (*R*^2^ = 0.8). Three donors were used in these studies. Experimental replicates are indicated in parentheses above each data set.

We next determined the effects of varying the concentration of VX-445 and VX-121 on K^+^ secretion to assess concentration dependence. As shown in Figure 3A, VX-445 stimulated K^+^ secretion at low-micromolar concentrations, and exhibited a steep concentration dependence between 1 and 10 μM (Figure 3C). Likewise, VX-121 stimulated K^+^ secretion at low-micromolar concentrations. However, VX-121 appeared more potent, producing visible responses at 0.3 and 1 μM (Figure 3B). As above, paxilline inhibited the K^+^ currents stimulated by VX-445 and VX-121 (Figure 3, A and B). Summary data for these studies are provided in Figure 3C. As the response to VX-121 approached saturation at 10 μM, we were able to fit these data to the Hill equation (Figure 3D), and obtained an apparent EC_50_ of 4.4 μM with a Hill coefficient of 3. In contrast, VX-445 did not saturate at 10 μM, and thus we were not able to obtain a reliable estimate of the EC_50_ for this molecule. We did not routinely go to the next higher half-log concentration (30 μM), as this is above the concentration of VX-445 achieved in plasma (C_max_ = 15 μM; ref. 38). Further, in 3 experiments in which we applied 30 μM VX-445, the subsequent addition of paxilline resulted in an increase in R_te_ of only 154 Ω∙cm^2^, whereas in 8 experiments carried out on the same day in which 10 μM VX-445 was added, the subsequent addition of paxilline increased R_te_ by 654 Ω∙cm^2^. As one possibility to explain this result is an overall decrease in R_te_, we did not pursue these higher concentrations further. Importantly, our results demonstrate that VX-445 stimulates K^+^ secretion in the range of concentrations known for VX-445 in both plasma and cells of patients with CF (see Discussion).

**Figure 4:**
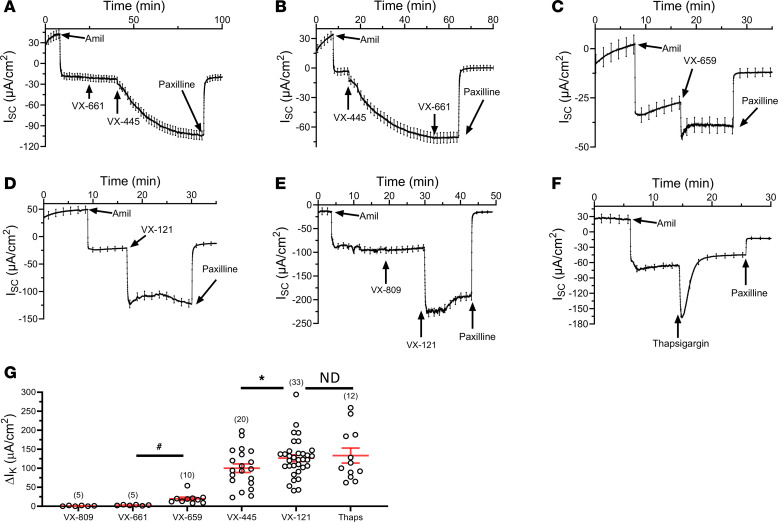
Effect of CFTR correctors on BK_Ca_ currents in F508del CFTR HBEs. Currents were recorded with a 125:5 mM K^+^ gradient (basolateral to apical) from uncorrected F508del HBEs. (**A**) Subsequent to amiloride, VX-661 (10 μM) failed to stimulate K^+^ secretion, while the further addition of VX-445 (10 μM) stimulated a paxilline-sensitive K^+^ secretory current. (**B**) After amiloride, VX-445 (10 μM) induced K^+^ secretion, whereas the addition of VX-661 (10 μM) failed to stimulate K^+^. (**C**) VX-659 (10 μM) induced a paxilline-sensitive K^+^ current. (**D**) VX-121 (10 μM) stimulated a paxilline-sensitive K^+^ current. (**E**) VX-809 (10 μM) failed to stimulate K^+^ secretion, while further addition of VX-121 (10 μM) induced an increase in K^+^ current. (**F**) Thapsigargin (1 μM) stimulated a transient K^+^ current. (**G**) Average ΔI_K_ values (mean ± SEM) for each compound evaluated (*P < 0.05; ^#^*P* < 0.01; ND, not different; 1-way ANOVA). Six donors were used in these studies. Experimental replicates are indicated in parentheses above each data set.

Next, we determined whether CFTR correctors stimulate K^+^ secretion across homozygous F508del CFTR HBEs. As shown in Figure 4A, following amiloride, the current SOC C1 corrector, VX-661 (10 μM), had no effect on K^+^ secretory current. However, subsequent addition of the current SOC C2 corrector, VX-445 (10 μM), stimulated a sustained K^+^ secretory current, akin to the response of WT CFTR HBEs. Further, initial addition of VX-445 (10 μM) induced a K^+^ secretory current that was not further increased by VX-661 (Figure 4B). Consistent with activation of an ionic conductance, VX-445 decreased R_te_ from 221 ± 7 Ω∙cm^2^ in the presence of amiloride to 146 ± 6 Ω∙cm^2^ (*P* < 0.001, *n* = 28), and this was increased to 491 ± 24 Ω∙cm^2^ (*P* < 0.001, *n* = 28) following paxilline addition. As in WT CFTR HBEs, VX-659 (10 μM) stimulated K^+^ secretion across F508del CFTR HBEs (Figure 4C). Similarly, the next-generation C2 CFTR corrector VX-121 (10 μM) stimulated a large, paxilline-sensitive K^+^ secretory current (Figure 4D). Again, this was accompanied by a decrease in R_te_ from 203 ± 9 Ω∙cm^2^ in the presence of amiloride to 140 ±4 Ω∙cm^2^ (*P* < 0.001, *n* = 28). Inhibition of BK_Ca_ by paxilline increased R_te_ to 441 ± 29 Ω∙cm^2^ (*P* < 0.001, *n* = 28), as expected. Finally, we determined whether VX-809 (lumacaftor), a first-generation C1 corrector, would affect transepithelial K^+^ currents across F508del CFTR HBEs. As shown in Figure 4E, VX-809 (10 μM) had no effect on K^+^ current, while the subsequent addition of VX-121 (10 μM) again stimulated a paxilline-sensitive K^+^ current. We used the peak response to thapsigargin (1 μM) as the gold standard for activation of BK_Ca_ (Figure 4F), as we have previously shown that thapsigargin stimulates K^+^ secretion across HBEs under the conditions used here (20). The average response of K^+^ secretion to each CFTR corrector across F508del CFTR HBEs is shown in Figure 4G. Based on these results, we conclude that C1 correctors fail to activate BK_Ca_, whereas the C2 CFTR correctors activate BK_Ca_ with a relative potency of VX-121 > VX-445 >> VX-659.

**Figure 5:**
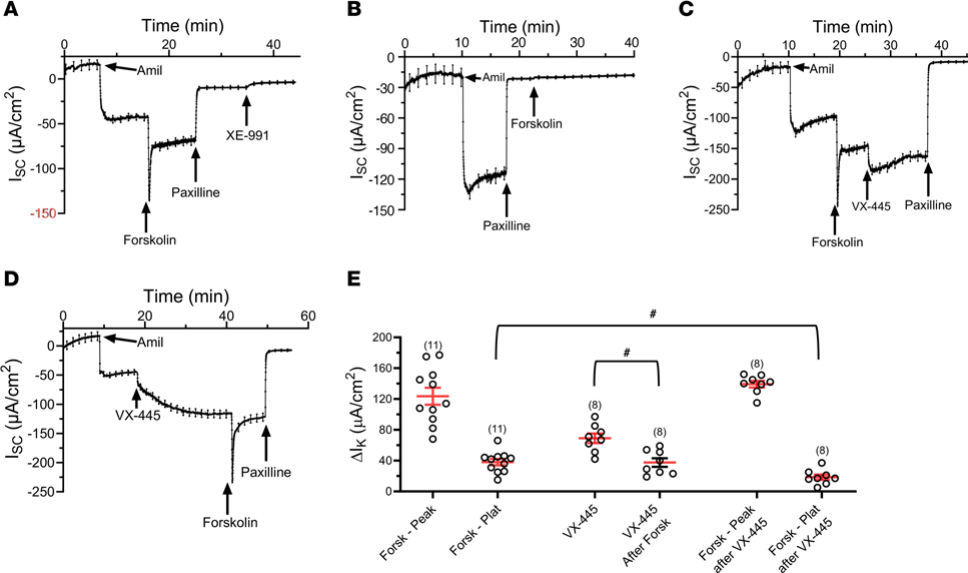
Effect of forskolin and VX-445 on BK_Ca_ currents in F508del CFTR HBEs. (**A**) Subsequent to amiloride, forskolin (10 μM) stimulated a paxilline-sensitive increase in K^+^ secretion, which is recognized as an initial transient spike followed by a sustained increase in I_sc_. Addition of XE-991 (10 μM) had little effect on the remaining current. (**B**) Subsequent to amiloride and paxilline, forskolin (10 μM) failed to stimulate K^+^ secretion. (**C**) Following forskolin, VX-445 (10 μM) induced a further increase in K^+^ secretion, which was paxilline sensitive. (**D**) VX-445 (10 μM) stimulated a sustained increase in K^+^ secretory current, which was further increased by forskolin. Subsequent addition of paxilline completely inhibited this K^+^ secretory current. (**E**) Average responses (mean ± SEM) to forskolin and VX-445 either alone or after addition of the previous agonist (^#^*P* < 0.01; unpaired ANOVA). Three donors were used in these studies. Experimental replicates are indicated in parentheses above each data set.

As CFTR is activated by cAMP/PKA, which also modulates BK_Ca_, it seems likely that CFTR and BK_Ca_ are simultaneously activated during cAMP-mediated agonist addition (39, 40). Thus, we determined whether forskolin stimulates K^+^ secretion across F508del CFTR HBEs and whether this affects the ability of VX-445 to stimulate K^+^ secretion. Initially, we verified that forskolin stimulates K^+^ secretion across F508del CFTR HBEs. As shown in Figure 5A, subsequent to amiloride, forskolin (10 μM) stimulated a rapid inward current followed by a sustained paxilline-sensitive plateau. As HBEs express Kv7.1, Kv7.3, and Kv7.5 (KCNQ) channels (35, 41, 42), which are also activated by cAMP/PKA, we determined whether these channels were contributing to the K^+^ secretory response elicited by forskolin. The pan-Kv7.X inhibitor XE-991 (10 μM) produced only a modest decrease in K^+^ secretory current induced by forskolin, suggesting that Kv7.X channels are not responsible for the K^+^ secretory current observed. To verify that the initial transient increase in K^+^ current was also due to BK_Ca_ activation, we used paxilline to inhibit the baseline K^+^ current induced by amiloride (Figure 5B), thereby validating that the inward current revealed by amiloride block of Na^+^ absorption was indeed due to K^+^ secretion. As shown in Figure 5B, preaddition of paxilline completely eliminated the forskolin response, verifying that both the peak and plateau currents were due to BK_Ca_ activation. Finally, we determined whether the VX-445 or forskolin responses were affected by the prior addition of the other compound. As shown in Figure 5C, VX-445 (10 μM) stimulated a further increase in K^+^ current subsequent to forskolin. However, in comparison with preaddition of VX-445 (Figure 5D), this response was decreased in magnitude (Figure 5E). Similarly, while prior addition of VX-445 (10 μM) did not affect the peak response to forskolin, the extent of the plateau phase was reduced (Figure 5, D and E).

**Figure 6:**
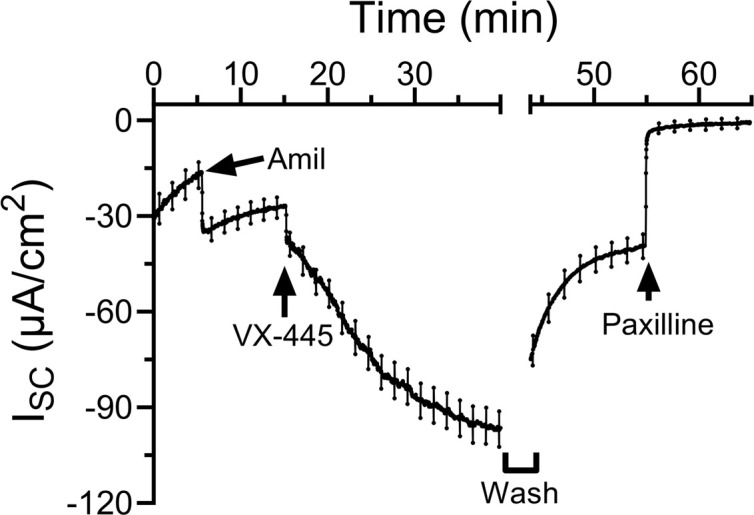
Effect of VX-445 on K^+^ current can be completely washed out in Ussing chambers. Following stimulation of K^+^ current by VX-445 (10 μM), both membranes were washed via 6 complete solution exchanges (noted by a break in recording). Following washout, the K^+^ current rapidly returned toward the pre–VX-445 current level. Subsequent addition of paxilline (10 μM) completely inhibited the remaining current. In 3 experiments, the average reduction in K^+^ current following washout of VX-445 was 90% ± 6%.

We next considered whether chronic exposure to VX-445 would affect BK_Ca_ currents. First, however, we determined whether the effect of VX-445 was readily reversible. This is important, as we could not maintain the filters in VX-445 during the experiment since this would simply recapitulate our demonstrated acute effects, even in the absence of a chronic effect. On the other hand, if VX-445 is readily reversible, any chronic effects may be lost when the filters are bathed in our apical/basolateral solutions. As shown in Figure 6, following stimulation of K^+^ current with VX-445 (10 μM), we carried out 6 complete solution exchanges of the apical and basal chambers (during a break in recording). Following this wash, BK_Ca_ short-circuit currents rapidly returned to pre-potentiated levels. Subsequent addition of paxilline (10 μM) inhibited the remaining current, as above. In 3 separate experiments, washout of VX-445 resulted in a reduction in current averaging 90% ± 6%. Thus, any effects of chronic exposure would be difficult to interpret, using these methods, and were not further pursued.

**Figure 7:**
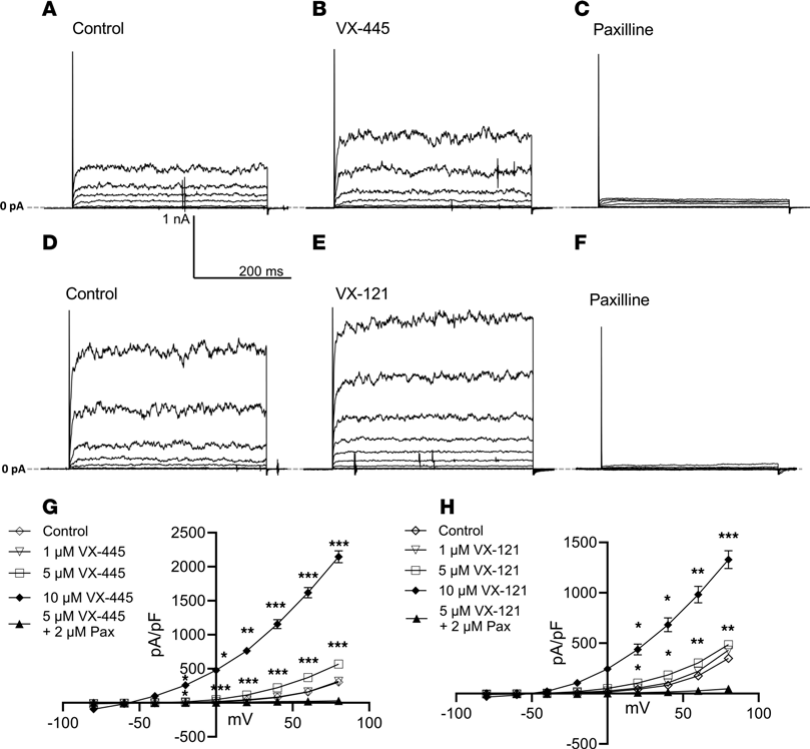
Effect of CFTR correctors on whole-cell BK_Ca_ currents heterologously expressed in HEK cells. (**A**) Control whole-cell recording. Voltage was stepped between –80 to +80 mV in 20 mV increments. (**B**) Whole-cell recording from the same cell following stimulation with 5 μM VX-445. (**C**) Paxilline (2 μM) completely blocked BK_Ca_ current. (**D**) Control whole-cell current recording. (**E**) Whole-cell current recording from the same cell following stimulation with 5 μM VX-121. (**F**) Paxilline (2 μM) completely blocked BK_Ca_ current. (**G**) Average whole-cell current-voltage (I-V) relationships (mean ± SEM) for control (*n* = 21) and 1 μM (*n* = 8), 5 μM (*n* = 14), and 10 μM (*n* = 11) VX-445 (*P < 0.05, **P < 0.01, ***P < 0.005; paired *t* test). Paxilline was added in the presence of VX-445 to illustrate lack of outward K^+^ currents in these cells when BK_Ca_ is blocked (*n* = 4). (**H**) Average whole-cell I-V relationships (mean ± SEM) for control (*n* = 14) and 1 μM (*n* = 6), 5 μM (*n* = 8), and 10 μM (*n* = 6) VX-121 (*P < 0.05, **P < 0.01, ***P < 0.005; paired *t* test). Complete inhibition by paxilline confirms a lack of additional VX-121–potentiated currents in this stable cell line.

The simplest explanation for our HBE results is that the C2 CFTR correctors VX-659, VX-445, and VX-121 directly activate apical membrane BK_Ca_. To assess this, we determined whether VX-445 and VX-121 potentiate BK_Ca_ during whole-cell patch-clamp recordings from HEK cells (HEK-BK) heterologously expressing the pore-forming α subunit of BK_Ca_ (αBK_Ca_). For these studies, the cell was clamped at –80 mV and pulsed in 20 mV increments to +80 mV. As shown for a single cell (Figure 7, A–C), following establishment of a stable baseline (Figure 7A), VX-445 (5 μM) induced a significant increase in outward current (Figure 7B) that was completely inhibited by paxilline (Figure 7C). Similar studies were carried out using VX-121. As shown for a single cell (Figure 7, D–F), VX-121 (5 μM) potentiated αBK_Ca_ currents, which were blocked by paxilline. The average current-voltage (I-V) relationships for control and 1 μM, 5 μM, and 10 μM VX-445, as well as paxilline, are shown in Figure 7G. Both 5 and 10 μM VX-445 significantly increased BK_Ca_ current density across the range of voltages tested. The average I-V relationships for 1 μM, 5 μM, and 10 μM VX-121 are shown in Figure 7H. Similarly to VX-445, our results show that 5 and 10 μM VX-121 significantly potentiated αBK_Ca_ current density.

**Figure 8:**
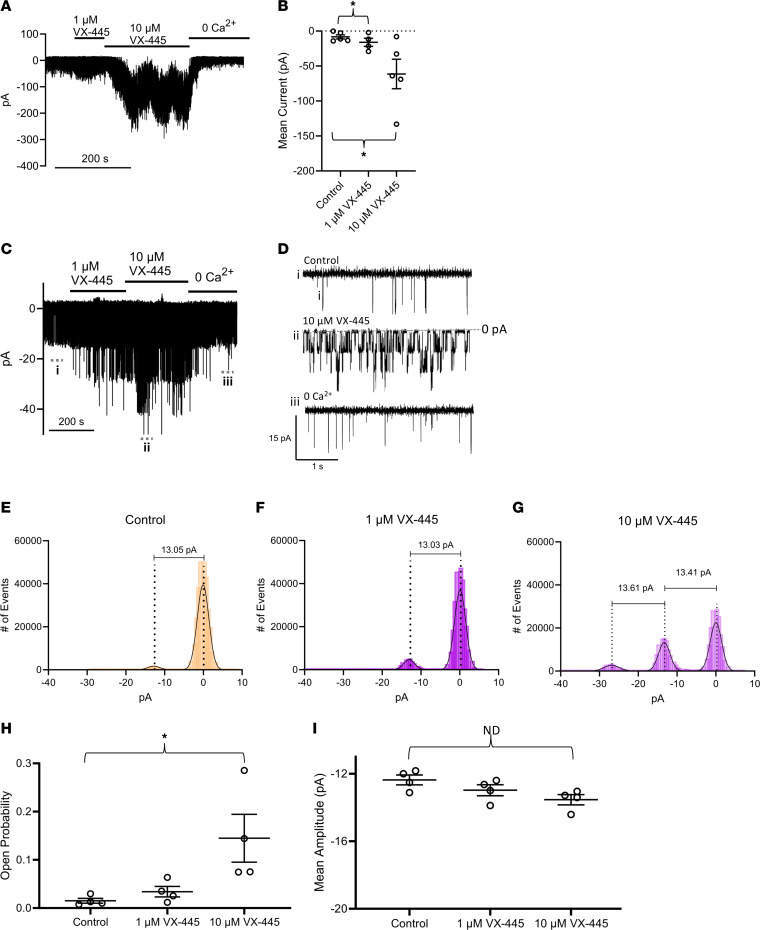
Effect of VX-445 in excised patches. Excised patch-clamp recordings were carried out in symmetric K^+^ and voltage-clamped to +40 mV. (**A**) Both 1 μM and 10 μM VX-445 increased current in a patch expressing many channels. The current was reversed by addition of 0 Ca^2+^. (**B**) Average mean current (pA, mean ± SEM) for excised patches containing numerous BK_Ca_ channels for control (*n* = 5) and 1 μM (*n* = 4) and 10 μM (*n* = 5) VX-445. (**C**) Both 1 μM and 10 μM VX-445 increased current in a patch expressing αBK_Ca_ such that individual channel openings could be observed. (**D**) Expanded view of trace shown in **C**, where individual BK_Ca_ channel open and closed events can be observed in magnified current traces corresponding to the parent trace (dashed lines and i, ii, and iii). (**E**–**G**) All-point histograms from the recording shown in **C** such that single-channel current amplitude can be determined, as indicated by the distance between the dotted lines placed at the peak of each curve, which represent a given open state. (**H**) Average P_o_ (mean ± SEM) calculated for 4 experiments, as shown in **C**. (**I**) Average single-channel amplitude for 4 experiments (*P < 0.05; paired ANOVA).

To demonstrate that the potentiation of αBK_Ca_ by VX-445 during whole-cell recording is a direct effect on the channel, we performed excised, inside-out patch-clamp recordings on HEK-BK cells. As shown in Figure 8A, excised patches often contained many αBK_Ca_ channels. In this case, mean currents were determined in the absence and presence of VX-445. As shown in Figure 8A, 1 μM VX-445 produced a small increase in total current, and this was further increased by 10 μM VX-445. Channel activity was dramatically reduced following perfusion of 0 Ca^2+^. As shown in Figure 8B, both 1 and 10 μM VX-445 produced a significant increase in mean current (*P* < 0.05). In additional inside-out patches, small numbers of channels were observed (<5), such that individual opening and closing events could be realized, thereby allowing us to determine both single-channel current amplitude (i) and open probability (P_o_). As shown for one experiment in Figure 8C, both 1 and 10 μM VX-445 increased channel P_o_, as evidenced by the increased frequency of discrete channel opening events (Figure 8, C and D). The average change in P_o_ for 4 patches is shown in Figure 8H, with 10 μM VX-445 inducing a 10-fold increase in P_o_. All-point histograms for the recording shown in Figure 8C during control (Figure 8E) and 1 μM (Figure 8F) and 10 μM (Figure 8G) VX-445 demonstrated no significant change in i (Figure 8I). In total, our patch-clamp studies demonstrate that the current SOC C2 corrector, VX-445, directly potentiates αBK_Ca_ via an increase in P_o_, likely explaining the K^+^ secretion observed across HBEs.

**Figure 9:**
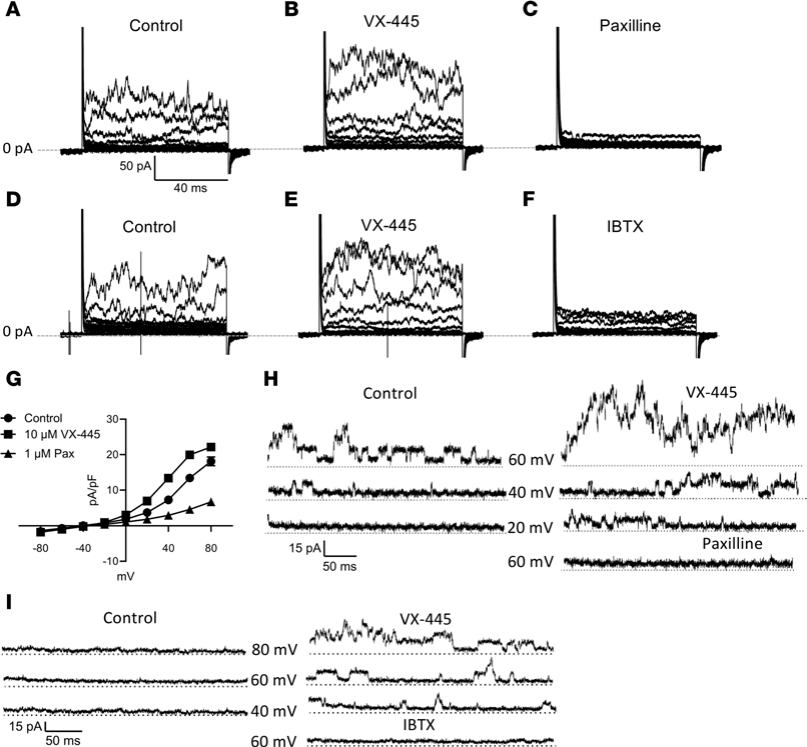
VX-445 potentiates paxilline- and IBTX-sensitive currents in WT CFTR HBEs. (**A**) Control whole-cell recording from primary HBEs during voltage steps from –80 to +80 mV in 20 mV increments. (**B** and **C**) Effect of VX-445 (**B**, 10 μM) and paxilline (**C**, 1 μM) on the cell shown in **A**. (**D**) Control whole-cell recording from primary HBEs. (**E** and **F**) This current was potentiated by VX-445 (**E**, 10 μM) and inhibited by IBTX (**F**, 300 nM). (**G**) Mean I-V (mean ± SEM) for control (squares), 10 μM VX-445, and 1 μM paxilline from 9 experiments. (**H**) Whole-cell recording from primary HBEs where individual single-channel openings can be observed at +20, +40, and +60 mV. Control traces (left) exhibit fewer channel openings when compared with those observed in the presence of 10 μM VX-445 (right) or when 1 μM paxilline was added to inhibit BK_Ca_ activity (bottom right). (**I**) Whole-cell recording from primary HBEs where individual single-channel openings cannot be observed at +40, +60, and +80 mV in control recordings (left). Addition of VX-445 (10 μM, right) induced channel activity, which was inhibited by paxilline (1 μM, bottom right).

Having verified potentiation of αBK_Ca_ in HEK-BK cells, we determined whether C2 correctors potentiate BK_Ca_ currents in primary undifferentiated, nonpolarized HBEs via whole-cell patch clamp. As shown in Figure 9A, HBEs exhibited an outwardly rectified current that was potentiated by VX-445 (10 μM; Figure 9B) and subsequently completely inhibited by paxilline (Figure 9C), verifying expression of BK_Ca_ and potentiation by VX-445. To further validate the identity of these VX-445–potentiated currents, we used another canonical blocker of the BK_Ca_ channel, IBTX. As shown in Figure 9F, IBTX inhibited the VX-445–potentiated K^+^ currents (Figure 9, D and E). The average I-V relationships for 9 experiments are shown in Figure 9G. Interestingly, some primary HBEs, surveyed by whole-cell patch clamp, had low-level BK_Ca_ expression, in which single-channel activity was observed (Figure 9, H and I). In Figure 9H, under control conditions (left array of traces), little or no channel activity was observed at +20 or +40 mV, while at +60 mV clear channel activity was seen. This is consistent with the voltage dependence of BK_Ca_ channels. However, in the presence of VX-445 (10 μM; right array of traces) multiple channel openings were observed at all voltages. Paxilline completely abrogated channel activity in this cell (Figure 9H, right bottom trace). In a separate cell (Figure 9I), no channel activity was observed at +40, +60, or +80 mV under control conditions (left array of traces). Subsequent addition of VX-445 (10 μM) induced individual channel events, which were silenced with 300 nM IBTX. These data validate BK_Ca_ potentiation in HBEs, which consist of several cell types that can potentially express BK_Ca_ (43, 44), including CFTR-expressing ionocytes (45).

**Figure 10:**
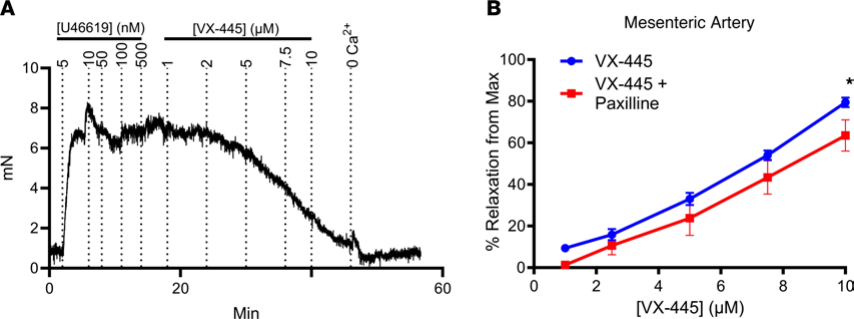
Effect of VX-445 on vasoreactivity in mouse mesenteric artery. (**A**) Recording of force in millinewtons (mN) over time from a single mesenteric artery showing preconstriction with the prostaglandin mimetic U46619 (1 × 10^−7^ to 5 × 10^−7^ M), after which the ability of increasing concentrations of VX-445 to induce vasorelaxation was assessed. We added 0 Ca^2+^ at the end to determine maximal vasorelaxation. (**B**) Average responses to VX-445 under control conditions (blue line, *n* = 10) and following preincubation with paxilline (10 μM) for 15 minutes (red line, *n* = 6). The effect of VX-445 was partially reversed by paxilline, verifying a role for BK_Ca_. *Statistical difference between VX-445 and VX-445 + paxilline by 2-way ANOVA with *P* < 0.003 by post hoc Holm-Šidák multiple-comparison test. Data are shown as mean ± SEM.

Our data demonstrate that C2 CFTR correctors directly potentiate BK_Ca_ channels, resulting in K^+^ secretion across WT and F508del CFTR–expressing HBEs. As BK_Ca_ is widely expressed throughout the body (46, 47), it is important to determine whether the current SOC C2 corrector, VX-445, modulates the function of additional tissues where BK_Ca_ is expressed. Initially, we determined the effect of VX-445 on vascular reactivity, as activation of BK_Ca_ hyperpolarizes vascular smooth muscle, resulting in vasorelaxation (48, 49). To assess the effect of VX-445 on vasoreactivity, mouse mesenteric arteries were preconstricted with the prostaglandin mimetic U46619 (1 × 10^−7^ to 5 × 10^−7^ M), and the ability of VX-445 to induce vasorelaxation was assessed. As shown in Figure 10A for a single mesenteric artery, following U46619-induced vasoconstriction, VX-445 induced vasorelaxation in a concentration-dependent manner. The average response is shown in Figure 10B (blue), with near-complete vasorelaxation achieved at 10 μM VX-445. This effect was partially attenuated by paxilline (Figure 10B, red), demonstrating VX-445 alters vasoreactivity in a BK_Ca_-dependent manner.

**Figure 11:**
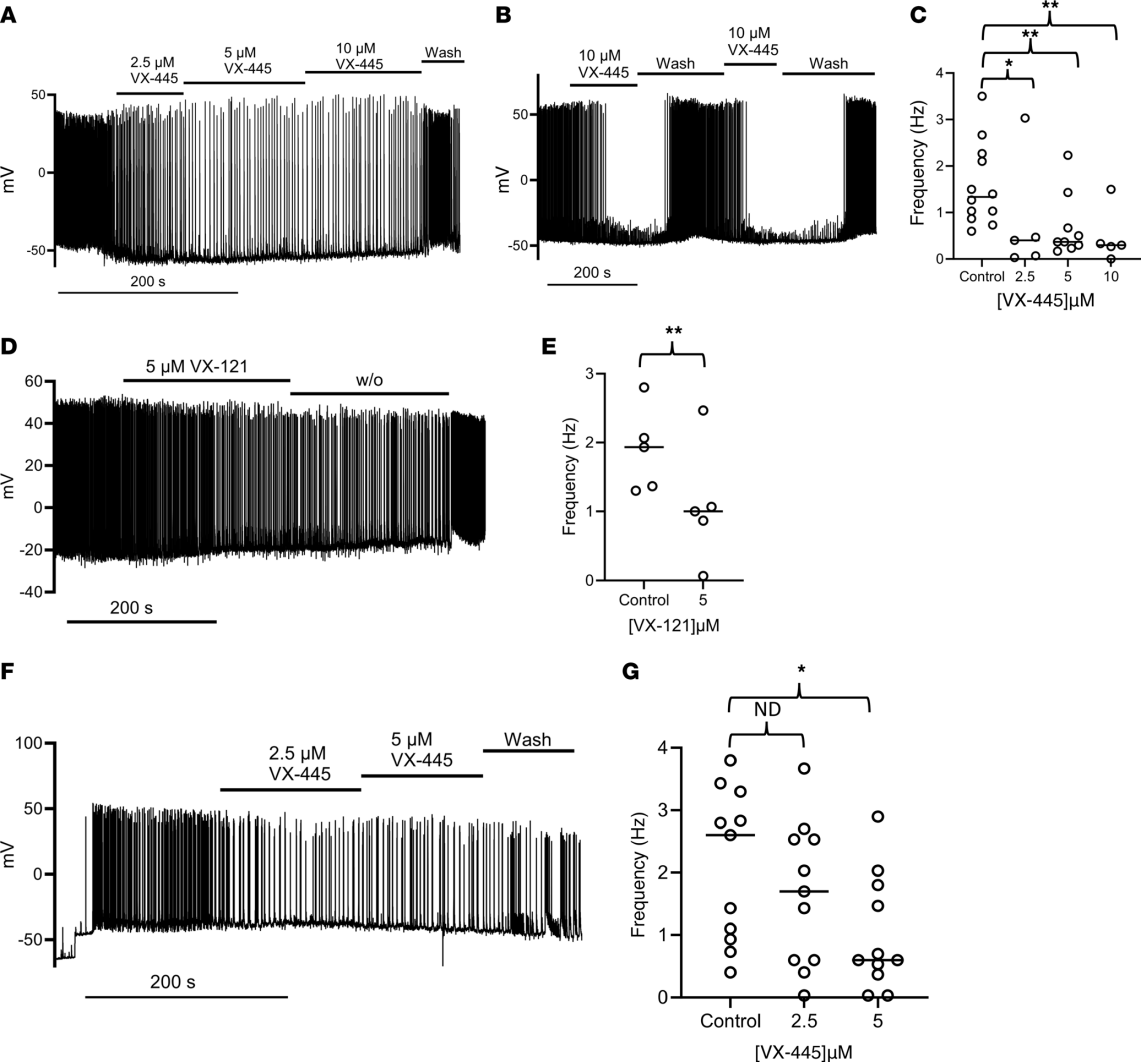
Effect of CFTR correctors on action potential firing in primary rat hippocampal and cortical neurons. (**A**) Effect of 2.5, 5, and 10 μM VX-445 on action potential firing in a spontaneously firing hippocampal neuron. (**B**) Hippocampal neuron demonstrating the reversible inhibition of action potential firing by 10 μM VX-445. (**C**) Average action potential firing frequency, in hertz, for experiments carried out as in **A** and **B** for control (*n* = 9) and 2.5 μM (*n* = 5), 5 μM (*n* = 9), and 10 μM (*n* = 5) VX-445 (*P < 0.05, **P < 0.01; paired *t* test). (**D**) Effect of VX-121 (5 μM) on a spontaneously firing hippocampal neuron. (**E**) Average action potential firing frequency for control and VX-121 (*n* = 5) (**P < 0.01). (**F**) Effect of VX-445 on action potential firing frequency in a primary cortical neuron. Action potentials were induced by current injection (step change in voltage). (**G**) Average action potential firing frequency (hertz) for control and 2.5 μM and 5 μM VX-445 in cortical neurons (*n* = 11 for all conditions, *P < 0.05; paired ANOVA).

In the nervous system, BK_Ca_ channels are a key component of the fast afterhyperpolarization, which is an important contributor to neuronal firing frequency (50, 51). Indeed, channelopathies involving both gain and loss of function of BK_Ca_ have been reported (52, 53). Thus, we determined whether VX-445 and VX-121 alter neuronal excitability. To accomplish this, current-clamp patch-clamp recordings were performed on primary E18 rat hippocampal and cortical neurons, and action potential firing frequency was monitored. As shown for 2 separate recordings from spontaneously firing hippocampal neurons (Figure 11, A and B), VX-445 induced a concentration-dependent decrease in action potential firing frequency, which was reversible upon washout. Indeed, as shown in Figure 11B, action potential firing frequency could be repeatedly inhibited by 10 μM VX-445. The average changes in firing frequency (in hertz) for 2.5 μM (*n* = 5), 5 μM (*n* = 9), and 10 μM (*n* = 5) VX-445 are shown in Figure 11C. As shown in Figure 11D, VX-121 (5 μM) similarly reduced action potential firing frequency in a primary hippocampal neuron, with the average change for 5 experiments shown in Figure 11E. Finally, we determined whether VX-445 would similarly alter the action potential firing frequency in primary cortical neurons to begin to assess the generalizability of our results. In contrast to hippocampal neurons, current injection was required to induce action potential firing in cortical neurons, under our recording conditions. As shown in Figure 11F, after current injection (delineated by the step change in voltage at the initiation of the trace), action potentials were observed. Subsequent addition of VX-445 (2.5 and 5 μM) induced a significant reduction in action potential firing frequency that was poorly washed out. The average change in firing frequency for 11 separate neurons is shown in Figure 11G. These results clearly demonstrate that VX-445 and VX-121 alter neuronal excitability, the implications of which are discussed below.

## Discussion

Over the past 15 years, the most significant advancement in CF therapeutics has been the development of CFTR potentiators and correctors (54–56). ETI has proven to be highly efficacious for patients with CF, resulting in diminished morbidity as well as vast improvements to their quality of life (18, 19). Key components of ETI are the C1 (VX-661; tezacaftor) and C2 (VX-445; elexacaftor) CFTR correctors, which partially restore the folding and hence trafficking of misfolded CFTR to the apical plasma membrane (15). VX-445 has also been shown to potentiate CFTR (57, 58). These C1 and C2 correctors exhibit distinct binding sites on CFTR, resulting in unique mechanisms of action (10, 11, 15, 55). Importantly, we demonstrate that C1 correctors fail to modulate BK_Ca_ activity (Figure 1D and Figure 4, A, B, and E), whereas C2 correctors strongly potentiate BK_Ca_, suggesting a binding site on BK_Ca_. VX-445 and VX-659 were developed simultaneously by Vertex Pharmaceuticals and have a similar scaffold (59), whereas VX-121 is notably distinct. Our results demonstrate that VX-121 is a more potent potentiator of BK_Ca_ than VX-445 and VX-659 in HBEs, and the potentiation response exhibits a faster onset (Figures 1–4). Interestingly, as clinical development has improved the efficacy of C2 correctors to rescue misfolded CFTR, so has the potency to activate the BK_Ca_ channel (VX-121 > VX-445 >> VX-659).

We recently demonstrated that potentiation of basolateral KCa3.l by DCEBIO (5,6-dichloro-1-ethyl-1,3-dihydro-2H-benzimidazol-2-one) further stimulates transepithelial Cl^–^ secretion across VX-445/VX-661–corrected primary F508del HBEs (35). This indicates the potential for a K^+^ conductance to be rate-limiting for Cl^–^ secretion. It has been proposed that potentiation of BK_Ca_ would similarly increase the electrochemical driving force for Cl^–^ exit across the apical membrane, such that BK_Ca_ may be an alternate pharmacological target in CF (26). Indeed, BK_Ca_ is important in maintaining ASL volume in the airway (26, 27). In airway epithelia, BK_Ca_ coassembles with its γ subunit (gene symbol: LRRC26) (60), as well the β_2_/β_4_ subunits. The cytokines IFN-γ and TGF-β, which are elevated in CF, both reduce functional apical expression of BK_Ca_ through downregulation of LRRC26 (27, 28). Furthermore, CF-related diabetes mellitus (CFRD) has been linked to a reduced expression of LRRC26 (61), suggesting that one possible explanation for the worse lung outcomes observed in CF patients with CFRD (62) is a reduction in apical BK_Ca_ expression. Previous modeling by Sandefur and colleagues (63) suggested a role for apical BK_Ca_ in K^+^ secretion, predicting an increased K^+^ secretion across the CF airway to balance Na^+^ absorption. In this regard, it has been shown that K^+^ is elevated above plasma in the ASL, being about 20–30 mEq, suggesting that human airways secrete K^+^ (6, 42).

Given the role BK_Ca_ plays in maintaining ASL volume, it is possible that the previously unrecognized ability of C2 CFTR correctors to potentiate BK_Ca_ may be providing CF HEMTs with additional clinical benefit. As CFTR and BK_Ca_ are both regulated by cAMP/PKA (Figure 5), it seems likely that these channels act in a concerted fashion to stimulate KCl secretion. In this way, the positive regulation of BK_Ca_ by VX-445 may help to negate the dehydrating effects of inflammation or CFRD at the air-liquid interface by increasing the P_o_ of available plasma membrane BK_Ca_. However, as reported in preliminary studies (64), our attempts to directly determine whether VX-445 potentiates cAMP-mediated Cl^–^ secretion across HBEs paradoxically demonstrated that VX-445 inhibits forskolin-mediated Cl^–^ secretion across both WT and F508del CFTR HBEs. This inhibition of Cl^–^ secretion was a result of VX-445 directly inhibiting the basolateral membrane KCa3.1 channel (our unpublished observations). Thus, any effects of BK_Ca_ potentiation may be masked by this additional effect on KCa3.1.

Our data demonstrate that the current SOC C2 corrector, VX-445, and the next-generation C2 CFTR corrector, VX-121, potentiate BK_Ca_ in the low-micromolar range (1–10 μM). Thus, it is important to consider whether these are clinically relevant concentrations in patients with CF. In this regard, clinical trial data have shown maximal (C_max_) and minimal (C_min_) plasma elexacaftor (VX-445) concentrations of 8.4–9.2 μg/mL (about 15 μM) and 4.0–5.4 μg/mL (about 6–9 μM), respectively (38, 65, 66). More recently, mean concentrations of VX-445 in cell lysates from CF patient nasal brushings have been found to range from 0 to 5,454 ng/mL (9 μM) (67). Thus, the low-micromolar effects we observe in HBEs and microvascular arteries may be directly relevant in a clinical setting. However, to our knowledge, the concentrations of CFTR correctors achieved in the brain are unknown. Given our results demonstrating effects of VX-445 and VX-121 on action potential firing frequency, this is an important unresolved question.

While the current HEMT has proven to be highly efficacious for improving lung function as well as quality of life for most patients with CF, it is important to point out that in a subset of patients, adverse events (AEs) have been reported (31, 68–71). Both symptomatic and asymptomatic hypertension has been a feature of clinical trials involving CFTR correctors, and in some cases therapy has been discontinued as a result (31, 32). In addition, individuals expressing the F508del allele have reported headache on ETI, which has also been reported in clinical trials of the clinically evaluated BK_Ca_ agonist BMS-204352 (73–76). As VX-445 alters vasoreactivity in our experiments, the effects of VX-445 on BK_Ca_ could potentially explain these AEs. However, it should be noted that we observed only a partial reversal of this effect with paxilline, suggesting that other ion channels may play a role in the observed effect. For example, in preliminary studies (64), we demonstrate that VX-445 inhibits members of the KCNN gene family, including KCa3.1 and KCa2.x channels. These channels are known to play a critical role in maintaining vascular reactivity (77, 78). Further, CFTR mutations have also been shown to affect smooth muscle contractility (79, 80), suggesting that the known potentiation effect of VX-445 on CFTR may also play a role in the effects observed. While we demonstrate a direct effect of VX-445 on BK_Ca_ and vascular reactivity, further studies are required to clarify the role of each of these conductances in the overall vascular response.

Critically, mental-status changes, which may result from changes in neuronal activity, have been reported in response to ETI (29–31, 33, 34, 81). Several patients have described their symptoms as “mental fogginess” and reported deficits to several aspects of cognition. In most of these patients, the onset of symptoms appeared 1 month after initiation of treatment. In one study, 2 patients discontinued treatment because of their reported AEs (33). Furthermore, as attempted suicide has been reported in patients on ETI, clinicians have recommended close monitoring (29). As patients with CF are predisposed to anxiety and depression (82), limiting these adverse mental-status effects relating to mood, motivation, or cognition is of paramount clinical importance. A recent meta-analysis of ETI trials failed to show a causal relationship between ETI therapy and depression-related symptoms, which the authors concluded were commensurate with the background epidemiology of patients with CF (82). These findings are not uncontested, however, and currently the implications of ETI therapy for mental-status AEs are highly disputed (83) and deserving of serious research effort (84).

While it has been speculated that CFTR correctors and/or potentiators may affect neuronal function (30, 31), this has not been directly demonstrated. As noted, BK_Ca_ channels play a critical role in regulating action potential firing via the fast afterhyperpolarization (51). Either pharmacological or genetic manipulation of BK_Ca_ function dramatically affects action potentials (51, 85). Herein, we demonstrate, for the first time to our knowledge, that the C2 CFTR correctors VX-445 and VX-121 directly affect action potential firing frequency in primary cultures of hippocampal and cortical neurons (Figure 11). Whether this effect of CFTR correctors can account for the mental-status changes reported by people with CF on ETI is purely a matter of speculation. However, given the critical role BK_Ca_ plays in neuronal action potential firing, we believe our results lay the critical foundation for future studies designed to further test this hypothesis.

Going forward, it may be both prudent and challenging to design the next class of CFTR correctors to avoid activation of the BK_Ca_ channel. In addition to being regulated by Ca^2+^ and voltage, the pore-forming α subunit of BK_Ca_ can also associate with 4 unique β subunits (β_1_–β_4_) as well as 4 γ subunits (γ_1_–γ_4_). Indeed, the association of αBK_Ca_ with β_2_/β_4_ and γ_1_ in HBEs results in the functional apical membrane K^+^ channel (26, 27). Further, the BK_Ca_ α subunit exhibits alternate splicing. In this regard, the stress axis–regulated insert (STREX) variant of BK_Ca_ is expressed by HBEs grown in depleted medium, such that its regulation by phosphorylation is affected (86). In this study, we verified activity of VX-445 and VX-121 on the α subunit of BK_Ca_. However, it remains to be determined whether regulatory subunits or splice variants play a role. With regard to accessory subunits, the β_1_ subunit is highly expressed in smooth muscle (87). In the CNS, expression of auxiliary subunits β_1_, β_2_, β_3b_, β_3c_, β_3d_, γ_1_, γ_3_, and γ_4_ has been reported (60). We demonstrate effects of C2 CFTR correctors on lung, arterial, and brain tissue, a tissue set encompassing most BK_Ca_ auxiliary subunits. Thus, while we cannot say how auxiliary subunits might affect the response of BK_Ca_ to CFTR correctors, we are confident that the presence of these subunits does not preclude this effect.

CFTR HEMTs represent a critical component of the CF therapeutic armament, allowing patients with CF to live well into adulthood. Herein, we demonstrate, for the first time to our knowledge, that the current SOC C2 CFTR corrector, VX-445, directly potentiates a distinct ion channel other than CFTR — BK_Ca_. Given the clinical benefit of these lifesaving drugs, we would emphasize 2 major points with respect to cross-reactivity with the BK_Ca_ channel. First, the potentiation of BK_Ca_ by VX-445 and VX-121 in F508del CFTR HBEs may represent a previously unrecognized mechanism of action resulting in clinical benefit due to potentiated K^+^, and hence Cl^–^ secretion. Second, while there is a correlation between the physiological effects of C2 correctors (Figures 10 and 11) and the AEs reported by patients with CF, a great deal of work remains to link our in vitro data presented herein to the AEs reported clinically. Nevertheless, given our data, the wide expression of BK_Ca_, and its clear link to disease following over- or underexpression (85), the proposal to target this channel in airway epithelia may be fraught with difficulties. Finally, given that VX-445 and VX-121 represent novel BK_Ca_ activators, these compounds could prove useful in dissecting the role of BK_Ca_ in a host of settings. Indeed, as VX-445 is an FDA-approved drug, it may be useful in a host of conditions in which BK_Ca_ potentiators have been proposed to have therapeutic utility (53, 73–76, 88, 89). Therefore, we feel it prudent, in the iterative spirit of drug development, that the structure of existing C2 correctors be interrogated to identify the precise pharmacophore responsible for BK_Ca_ versus CFTR regulation, in order to design more selective compounds for CF.

## Methods

### Sex as a biological variable.

Sex was not considered a variable in this work, as the authors were unaware of the sex of human donor tissue.

### Cell culture.

HEK293 cells expressing αBK_Ca_ were provided by Heike Wulff (University of California, Davis, Davis, California, USA) and cultured as in our previous work (90).

Primary HBEs were provided by the University of Pittsburgh human airway cells and tissue core and cultured using the Vertex method (91). Our studies were carried out on WT CFTR HBEs from 9 donors and homozygous F508del/F508del CFTR HBEs from 6 donors. HBEs were plated on Costar Transwell permeable supports (0.4 μM pore size, 6.5 mm insert, polyester membrane; Corning) and grown at an air-liquid interface for more than 5 weeks in HBE differentiation medium containing 2% Ultroser G. Basolateral medium was replaced 3 times per week. Two days before electrophysiological studies, accumulated mucus was removed from the apical membrane using 70 μL of 37°C PBS for 30 minutes. For whole-cell patch-clamp studies, HBEs were maintained in BronchiaLife airway medium (LS-1047, Lifeline Cell Technology) and plated onto poly-d-lysine–coated coverslips (see below).

### Ussing chamber short-circuit current measurements.

Costar Transwell inserts were mounted in a modified Ussing chamber (P2300, Physiologic Instruments) and the monolayers continuously short-circuited (VCC MC8, Physiologic Instruments) by forcing of the transepithelial voltage to 0 mV. R_te_ was monitored by application of a 2 mV pulse every 90 seconds. For measurements of transepithelial K^+^ secretion (I_K_) the basolateral solution contained (in mM): 120 K-gluconate, 25 NaHCO_3_, 3.3 KH_2_PO_4_, 0.8 K_2_HPO_4_, 1.2 MgCl_2_, 4 CaCl_2_, and 10 glucose. For the apical solution, Na-gluconate was substituted for K-gluconate, thereby creating a 125:5 mM basolateral-to-apical K^+^ concentration gradient. CaCl_2_ was used at 4 mM to account for the Ca^2+^ buffering capacity of gluconate. The pH of the solution is 7.4 when gassed with 95% O_2_/5% CO_2_. In a subset of experiments, the basolateral membrane was permeabilized with nystatin (200 μM), as previously described (37). The limited Cl^–^ in these solutions (~10 mEq) relieves cell swelling associated with the limited permeability of the nystatin pore to Cl^–^ (92).

Experiments were carried out at 37°C, and compounds were added cumulatively following establishment of a new stable current response. Paxilline, iberiotoxin, and amiloride were added to the apical membrane, while all other compounds were added to both membranes owing to their lipophilic nature. In all experiments, 10 μM amiloride was used to inhibit sodium absorption. ΔI_K_ was calculated as the difference between the baseline current after amiloride inhibition and the peak response to the agonist.

### Rat hippocampal and cortical neuron dissection and primary neuron culturing.

Hippocampal and cortical neurons were dissected from E18 Long-Evans rat embryos as previously described (93). Dissociated neurons (1 × 10^5^ cells per well) were plated on acid-washed 12 mm coverslips coated overnight with poly-d-lysine (high molecular weight 20 mg/mL) and laminin (3.4 mg/mL). Neurons were cultured in Neurobasal medium (Invitrogen) supplemented with 2% B27 (Invitrogen), penicillin and streptomycin (100 U/mL and 100 mg/mL, respectively), and 2 mM glutamine. For hippocampal neurons, 40% of the medium was replaced every 4 days, whereas for cortical neurons, 50% of the medium was replaced daily. Patch-clamp studies were carried out 1–3 weeks after plating. All animal studies were approved by the University of Pittsburgh Institutional Animal Care and Use Committee (protocol 22051190).

### Mesenteric arteries.

Wire myography experiments were conducted similarly to those published (94–96). Male C57BL/6 mice between 10 and 12 weeks of age were purchased from The Jackson Laboratory. Mice were euthanized by CO_2_ asphyxiation, and mesenteric arteries (MAs) were isolated and cut into 2 mm segments. MAs were placed in a physiological salt solution (PSS) containing (in mM): 0.026 EDTA, 119 NaCl, 5.5 d-glucose, 25 NaHCO_3_, 4.7 KCl, 1.17 MgSO_4_, 1.18 KH_2_PO_4_, and 2.5 CaCl_2_. PSS was brought to a pH of 7.4 by bubbling with 95% O_2_/5% CO_2_ at 37°C. MAs were mounted on a wire myograph (Multiple Myograph Model 620 M, Danish Myotechnology) using 25 μm–diameter tungsten wire and allowed to rest for 30 minutes in PSS. MAs were incrementally stretched to a tension equivalent to 80 mmHg of physiological pressure. MA viability was tested via the addition of potassium (60 mM) in PSS for 5 minutes, followed by 3 washes with PSS. After a 30-minute rest period, MAs were incubated for 15 minutes with either paxilline (10 μM) or control buffer (0.1% DMSO). Subsequently, MAs were constricted with the prostaglandin mimetic U46619 (1 × 10^−7^ to 5 × 10^−7^ M) for 4 minutes per concentration to induce maximal constriction before vasodilator treatment. After vessels reached maximal constriction, a cumulative concentration-response curve for VX-445 was conducted (1, 2.5, 5, 7.5, and 10 μM for 15 minutes per concentration). Subsequently, Ca^2+^-free PSS containing 1 × 10^−6^ sodium nitroprusside was added to determine maximal relaxation. Data were recorded on Lab Chart Software (AD Instruments), and relaxation percentage was normalized to the change in maximal constriction via U46619 and maximal dilation via Ca^2+^-free PSS.

### Whole-cell patch-clamp electrophysiology.

To investigate C2 corrector activity on αBK_Ca_, HEK cells expressing the αBK_Ca_ channel were plated onto poly-l-lysine–coated (0.01%; MilliporeSigma) glass coverslips 1 day before patch-clamp analysis. WT CFTR–expressing HBEs were used up to 72 hours after plating. For whole-cell experiments, pipettes were filled with a solution containing (in mM): 145 K-gluconate, 10 EGTA, 7.5 CaCl_2_, 2 MgCl_2_, 3 mM NaATP, and 10 HEPES. The bath solution contained (in mM): 140 K-gluconate, 5 KCl, 1.0 MgCl_2_, 10 HEPES, and 2 CaCl_2_.

For patch-clamp studies, an Axon 200B amplifier (Axon Instruments) in conjunction with Clampex data acquisition software (version 9.2, Axon Instruments) was used to capture recordings with low-pass Bessel filtering set at 2 kHz and a digitization rate of 10 kHz (1.48 MB/min). Recordings were analyzed using the relevant tools within Clampfit (version 9.2, Axon Instruments). Borosilicate glass electrodes (1.65 mm outer diameter; World Precision Instruments) were pulled with a Narishige puller (model PP-830). After fire polishing with a World Precision Instruments microforge (MF-200), pipettes had a resistance of 2–3 MΩ. A peristaltic pump (Minipuls 3, Gilson) was used to continuously perfuse bath solution (2.3 mL/min) to which various pharmacological agents were added. I-V relationships were determined via a pulse protocol involving a 400-millisecond voltage pulse from –80 to +80 mV flanked by a 50-millisecond pulse to a holding potential of –80 mV. The interpulse interval was 100 milliseconds. The middle 300 milliseconds of each trace was selected to avoid any influence of capacitive transients, and average currents were calculated for each voltage via the I-V tool in Clampfit. To control for cell size, whole-cell currents were normalized to cell capacitance.

For recordings from primary hippocampal and cortical neuronal cultures, cells were used between 1 and 3 weeks after being seeded on glass coverslips. Neuronal activity was recorded in current-clamp mode using a pipette solution containing (in mM): 140 K-gluconate, 0.5 CaCl_2_, 2 MgCl_2_, 1 EGTA, 10 HEPES, 2 NaATP, and 0.2 NaGTP, while the bath solution contained (in mM): 115 NaCl, 25 NaHCO_3_, 25 KCl, 2 CaCl_2_, 1 MgCl_2_, 10 glucose, and 10 HEPES (pH = 7.2 via KOH).

### Excised patch-clamp experiments.

For excised, inside-out patch-clamp recordings, the pipette solution contained (in mM): 145 K-gluconate, 5 KCl, 1.0 MgCl_2_, 10 HEPES, and 1 CaCl_2_, while the bath solution contained (in mM): 145 K-gluconate, 5 KCl, 1 EGTA, 1 HEPES, and 2 MgCl_2_. When appropriate, sufficient CaCl_2_ was added to obtain the desired free Ca^2+^ concentration, as previously described (97, 98). Solutions were adjusted to a pH of 7.2 with KOH. The zero current level was determined by addition of a 0 Ca^2+^ solution (zero added Ca^2+^ plus 1 mM EGTA), typically at the end of the experiment. Clampex data acquisition software (version 9.2, Axon Instruments) was used to capture recordings with the same filtering and digitization rates as above. For excised patch experiments, recordings were acquired in the gap-free continuous recording mode in which the membrane was held at +40 mV. Recordings were chosen for analysis that had no more than 5 easily recognized channel levels indicated by discrete opening and closing events. After adjustment of the baseline to the zero Ca^2+^ current levels in an interval with no channel activity, the single-channel search tool within Clampfit was used to identify channel events. P_o_ and single-channel amplitude (i) were then automatically calculated by Clampfit, once a histogram of events was produced and fit to a Gaussian distribution.

### Chemicals.

NS1619 (HY-12496), paxilline (HY-N6778), XE-991 (HY-108577), VX-445 (HY-111772), VX-661 (HY-15448), and VX-809 (HY-13262) were obtained from MedChemExpress. Thapsigargin (T9033), iberiotoxin (I5904), nystatin (N6261), and amiloride (A7410) were obtained from MilliporeSigma. Forskolin (F-9929) was obtained from LC Laboratories. VX-659 (2204245-48-5) was obtained from Stordsynthesis. VX-121 was synthesized by Kalexsyn. Ultroser G (NC1700979) was obtained from Pall Life Sciences. All other unspecified reagents were from MilliporeSigma.

### Statistics.

All data are presented as means ± SEM, where *n* indicates the number of filters or patch-clamp recordings. We assessed whether the data were normally distributed using both the D’Agostino and Pearson omnibus normality test and the Shapiro-Wilk normality test in GraphPad Prism (v10.1.0). Comparisons between 2 experimental maneuvers within an experiment were assessed for significance using a paired *t* test. Significance of differences between experiments was determined by an unpaired *t* test. Where deviations from the control mean were expected in 1 direction, such as in those experiments involving before and after measurements of BK_Ca_ potentiation in response to drug, a 1-tailed *t* test was used. When there was no expectation for the deviation from the mean in either direction, as in the case for comparison of ∆I_K_ between the effects of VX-445 and VX-121, a 2-tailed *t* test was used. Significance of differences between multiple experimental maneuvers within an experiment was determined by a 1-way ANOVA followed by a Tukey’s honestly significant difference post hoc test. Comparison between control and VX-445–induced vasoreactivity was evaluated by a 2-way ANOVA followed by a post hoc Holm-Šidák multiple-comparison test. All statistical analysis was carried out using GraphPad Prism (v10.1.0). Values of *P* less than 0.05 are considered statistically significant and are reported.

### Study approval.

All animal studies were approved by the University of Pittsburgh Institutional Animal Care and Use Committee (protocols 22051190 and 23063078).

### Data availability.

All data values from which a mean could be obtained have been provided in a Supporting Data Values tabulated spreadsheet organized by figure panel.

## Author contributions

AKA performed electrophysiological recordings and data analysis, made the initial discovery, contributed to experimental design, and prepared the manuscript. ST and ACS carried out vasoreactivity studies in mesenteric arteries, as well as manuscript preparation. MMM, as previous head, and JS, as current head, of the human airway core at the University of Pittsburgh contributed HBEs. ZPW contributed neurons for electrophysiological recordings. Ussing chamber experiments were carried out in the laboratory of MBB, who contributed to experimental design and manuscript preparation. RJB contributed to experimental design, as well as manuscript preparation. DCD contributed to all aspects of this work, including experimental design, electrophysiological recordings, data analysis, and manuscript preparation.

## Supplementary Material

Supporting data values

## Version 1 07/02/2024

In-Press Preview

## Version 2 08/15/2024

Electronic publication
